# Novel Anti-tumor Strategy for Breast Cancer: Synergistic Role of Oleuropein with Paclitaxel Therapeutic in MCF-7 Cells

**DOI:** 10.2174/0118715206284107231120063630

**Published:** 2024-01-24

**Authors:** Gamze Yılmaz, Filiz Özdemir

**Affiliations:** 1 Department of Biochemistry, Faculty of Pharmacy, Anadolu University, Eskişehir, Turkey

**Keywords:** Oleuropein, paclitaxel, MCF-7 cell line, breast cancer, oxidative stress, antioxidant

## Abstract

**Background:**

The side effects of conventional therapeutics pose a problem for cancer treatment. Recently, combination treatments with natural compounds have attracted attention regarding limiting the side effects of treatment. Oleuropein is a natural polyphenol in olives that has antioxidant and anticancer effects.

**Objectives:**

This study aimed to investigate the oxidative stress effect of a combination of Paclitaxel, a chemotherapeutic agent, and Oleuropein in the MCF-7 cell line.

**Methods:**

The xCELLigence RTCA method was used to determine the cytotoxic effects of Oleuropein and Paclitaxel in the MCF-7 cell line. The Total Oxidant and Total Antioxidant Status were analyzed using a kit. The Oxidative Stress Index was calculated by measuring Total Oxidant and Total Antioxidant states. The levels of superoxide dismutase, reduced glutathione and malondialdehyde, which are oxidative stress markers, were also measured by ELISA assay kit.

**Results:**

As a result of the measurement, IC_50_ doses of Oleuropein and Paclitaxel were determined as 230 µM and 7.5 µM, respectively. Different percentages of combination ratios were generated from the obtained IC_50_ values. The effect of oxidative stress was investigated at the combination rates of 10%, 20%, 30%, and 40% which were determined to be synergistic. In terms of the combined use of Oleuropein and Paclitaxel on oxidative stress, antioxidant defense increased, and Oxidative Stress Index levels decreased.

**Conclusion:**

These findings demonstrate that the doses administered to the Oleuropein+Paclitaxel combination group were lower than those administered to groups using one agent alone (*e.g*. Paclitaxel), the results of which reduce the possibility of administering toxic doses.

## 1 INTRODUCTION

Cancer is the disease with the highest mortality rate worldwide after cardiovascular diseases. Breast cancer, distinguished for its prevalence and fatality, stands as the most widespread form of cancer among women [1]. In the year 2020, it emerged as the most frequently diagnosed type of cancer in the female population [2]. The complex process involved in the development of breast cancer is revealed through distinct stages including initiation, progression, angiogenesis, and metastasis [3, 4]. Factors such as microenvironmental changes, cytokines, immunosuppression, and oxidative stress have been reported to play a role in the development and progression of cancer [5, 6]. Recent studies have directed their attention towards the impact of reactive oxygen species in the origination and progression of cancer.

Reactive oxygen species (ROS) are by-products produced continuously by cells during cellular metabolism. The balance between antioxidants and oxidants is important for maintaining redox homeostasis. Inadequate antioxidant defense and shifting of the balance to the side of oxidants triggers the formation of oxidative stress. Oxidative stress plays a role in the development of neurodegenerative disorders, aging, cataracts, diabetes mellitus, rheumatoid arthritis, cardiovascular diseases, respiratory diseases, and various types of cancer [7, 8]. High levels of ROS can cause DNA damage, mutation, chromosomal abnormalities, cancer initiation, increased cell proliferation, and activation of metastasis mechanisms, thus, resulting in cancer progression [9]. In addition, if the ROS level rises above the required amount, it can cause significant damage to the cell, such as mitochondrial membrane disruption, lipid peroxidation, and changes in secondary protein structures [10]. The combined use of chemotherapy, radiotherapy, and surgery in cancer treatment can have positive effects [11]. However, the increasing number of breast cancer cases and mortality rates in recent years have emphasized the need to develop more effective treatment methods [12]. Given the large number of reports linking reactive oxygen species to almost every step of tumor formation, several authors have suggested that ROS-generating and scavenging systems are potential targets for cancer therapy [13, 14]. Most chemotherapeutic drugs aim to kill cells by promoting ROS production. While triggering the death mechanisms in cancer cells, this condition also leads to the damage of normal cells.

In recent years, anthracycline- and taxane-class chemotherapeutic drugs have been widely used for the treatment of breast cancer. Paclitaxel (PTX), a taxane class of drugs, is an anticancer agent that plays an active role in tumor immunity and is used in the treatment of breast cancer [15]. PTX prevents the separation of microtubules by stimulating the union of tubulin with microtubules. It stops cell cycle progression and prevents mitosis [16]. PTX is known to induce apoptosis in the G1 and S phases. It stimulates both apoptosis and necrosis in the G2/M phase, triggers the formation of ROS, and changes mitochondrial membrane permeability [17, 18]. PTX is an effective anticancer drug; however, it has some side effects. These include hypersensitivity, damage to the peripheral nerves, and pain [19, 20]. In addition to the lethal effects of chemotherapeutic agents on cancer cells, the damage caused to normal cells is very high [21, 22].

The traditional Mediterranean diet is characterized by a low consumption of red meat and a high consumption of plant-derived foods [23]. Epidemiological studies have shown that cancer incidence rates in the Mediterranean countries are lower than those in other countries [24-26]. Low cancer incidence and mortality rates have been associated with olives and olive oil, which are widely consumed in Mediterranean countries. Products obtained from olive trees have attracted attention owing to their protective effects against cancer and many other diseases [27, 28]. In particular, olive leaves are rich in polyphenols, which inhibit the progression of cancer. Polyphenols can control the activity and expression of many of the target genes involved in the formation of cancer cells and can also affect the proliferation of tumor cells [29, 30]. Natural polyphenols can clear ROS produced during normal metabolism or that have increased due to external sources owing to their antioxidant effects [31]. Polyphenols also have pro-oxidant effects. As a result of these, they create a cytotoxic effect by causing excessive ROS production in cancer cells and can stimulate the death of cancer cells by increasing oxidative stress [32]. Oleuropein (OLE), a polyphenol and the main precursor for secoiridoids synthesis is the source of the antioxidant effects of olives and olives oils [33, 34]. OLE has beneficial health effects, including anticancer, antioxidant, antidiabetic, cardioprotective, and neuroprotective effects [35, 36]. OLE has low toxicity to normal cells, and its ability to affect cancer cells in different ways makes it a remarkable candidate for cancer therapy [37, 38].

Therefore, this study aimed to investigate the combined use of natural compounds and chemotherapeutic agents. Within the scope of our study, we determined the effects of OLE, which has strong antioxidant and anticancer properties, and PTX, a chemotherapeutic drug, both separately and in combination, on oxidative stress markers in breast cancer cells. The cytotoxic effects and IC_50_ doses of OLE and PTX were determined in MCF-7 breast cancer cells. The effects of OLE, PTX, and their combination on Total Oxidant Status (TOS) and Total Antioxidant Status (TAS) levels in the MCF-7 cell line for 24 hours were examined. The Oxidative Stress Index (OSI) was calculated using the TAS and TOS values determined. The effects of OLE, PTX, and their combinations on the activity of oxidative stress markers, such as Superoxide Dismutase (SOD), Glutathione (GSH) and Malondialdehyde (MDA), were measured.

## 2 MATERIALS AND METHODS

### 2.1 Materials

An MCF-7 cell line was obtained from the German Collection of Microorganism and Cell Culture (DSMZ, Leibniz Institute, Germany). High-glucose Dulbecco’s Modified Eagle’s Medium (DMEM), Fetal Bovine Serum (FBS), Trypsin-EDTA solution, penicillin/streptomycin solution, dimethyl sulfoxide (DMSO), OLE and PTX were purchased from Sigma Aldrich (St. Louis, MO, USA). TOS and TAS kits were purchased from Mega TIP (Gaziantep, Turkey). SOD, GSH, and TBARS Assay kits were purchased from Cayman (Michigan, USA).

### 2.2 Cell Culture

MCF-7 cells were maintained in DMEM containing 10% FBS and 1% penicillin/streptomycin in a humidified incubator of 95% air and 5% CO_2_ at 37°C.

### 2.3 Preparation of Concentration

Stock solutions of OLE and PTX were prepared in DMSO. The stock solution was stored at -20°C. Before the experimental studies, the working solution was prepared using a cell medium. Concentrations of 10, 25, 50, 100, 200, 300, and 400 µM were prepared to detect the IC_50_ of OLE. The concentration was prepared in the ranges of 5, 10, 15, 20, 25, 50, and 80 µM to detect the IC_50_ of PTX. The cytotoxic effects of substances on cells were determined by the xCELLigence Real-Time Cell Analyzer (RTCA) (ACEA Biosciences Inc., Roche, Germany) method. Untreated cells were used as the controls. All treated and untreated cells were incubated for 24 and 48 hours for use in experimental studies.

### 2.4 Cytotoxicity-proliferation Assay

The experiments were performed using the xCELLigence RTCA system at 37°C in a 5% CO_2_ environment. A 16-well E-plate was used to quantify the cytotoxic response of MCF-7 cells in real-time. MCF-7 cells were seeded on an E-plate at a density of 8x10^3^ cells/well and were kept in the incubator for 24 hours. At the end of the 24-hour incubation period, the concentrations of OLE (10, 20, 50, 100, 200, 300, 400 µM) and PTX (5, 10, 15, 20, 25, 50, 80 µM) were added to the E-plate wells and were kept in the incubator for 24 and 48 hours. Impedance was recorded at 15-minute intervals for experiments. IC_50_ values were determined using RTCA software (ACEA Biosciences, version number 2.0) using the cell index data.

### 2.5 Combined Drug Effect Analysis

For the drug combination experiments, different percentages of combinations were created with IC_50_ doses of OLE and PTX. MCF-7 cells were treated with combination ratios of 100%, 75%, 50%, 40%, 30%, 20%, and 10% generated with the determined IC_50_ doses of PTX and OLE. Analysis of dose-effect relationships was performed after measurement of cell growth using xCELLigence RTCA. Potential synergistic or antagonistic effects were calculated using CompuSyn Software (Cambridge, UK). To confirm that OLE and PTX were synergistic, we employed the “Chou-Talalay” method, which is derived from the mass-action law principle [39, 40]. Combination Index (CI) values and graphs were generated. The synergistic and antagonist effects of the drugs were determined according to the result of this.

### 2.6 Cell Lysate Preparation

Cell lysates for the TOS, TAS, SOD, GSH, and MDA assays were prepared according to the following method: MCF-7 cells were seeded on 6-well plates at a density of 7x10^5^ cells/well and kept in the incubator for 24 hours. At the end of the 24-hour incubation period, OLE, PTX IC_50_ doses, and synergistic effective combination groups were applied to the wells. At the end of the 24-hour incubation period, the adherent cell was washed by centrifugation at 1600 rpm for 6 minutes at 4°C. All the samples were placed on ice and a homogenizer was used to disrupt the pellet. The supernatant from centrifugation, which took place after lysis and lasted for 20 minutes at 4°C, was used for the experiment. The supernatant was stored at -20°C until used for measurements.

### 2.7 Determination of TOS/TAS Levels and OSI

#### 2.7.1 TOS Levels

The Rel Assay Diagnostics assay kit (KM211360) was used to determine the activity of TOS. This method provides general information about the total oxidant levels in cells. The oxidants in the samples oxidize the ferrous ions to ferric ions. The color change caused by the ferric ions in the acidic environment was measured using a spectrophotometer. The results were determined according to the kit instructions, using cell lysates prepared as described above. The absorbance was recorded at 530 nm using a spectrophotometer (Shimadzu UV-16A, Japan). The results were expressed as µmol H_2_O_2_ Equiv/L [41].


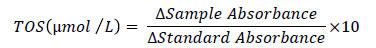


#### 2.7.2 TAS Levels

The Rel Assay Diagnostics assay kit (KM21123A) was used to determine the activity of TAS. This method provides general information about the total antioxidant levels in cells. The antioxidants in the samples reduce the dark blue-green 2,2'-azino-bis (3-ethylbenzothiazoline-6-sulfonic acid) (ABTS^+^) radical to a colorless form. Results were determined according to the kit instructions using cell lysates prepared as described above. Absorbance was recorded at 660 nm using a spectrophotometer. The results were expressed as µmol Trolox Equiv/L [42].


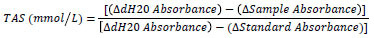


#### 2.7.3 Determination of OSI

To determine the oxidative balance status, the OSI was determined from the TOS and TAS values using the following formula. The TAS value was converted to µmol/L before calculating the OSI:


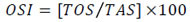


### 2.8 Determination of SOD

The Cayman Superoxide Dismutase Assay Kit (706002) was used to determine SOD activity. Results were determined according to the kit instructions using cell lysates prepared as described above. Absorbance was recorded at 450 nm using ELISA (BioTek ELx800, US).

### 2.9 Determination of GSH

The Cayman Glutathione Assay Kit (703002) was used to determine GSH activity. Results were determined according to the kit instructions using cell lysates prepared as described above. Absorbance was recorded at 405 nm using ELISA.

### 2.10 Determination of MDA

The Cayman TBARS Assay Kit (10009055) was used to determine MDA activity. Results were determined according to the kit instructions using cell lysates prepared as described above. Absorbance was recorded at 540 nm using ELISA.

### 2.11 Statistical Analysis

Three replicates of each experiment were performed. Results were expressed as mean ± standard error (SEM). Statistical analyses were performed using GraphPad Prism 9.5.0 (GraphPad Software Inc., California). Statistical differences between groups were determined using one-way ANOVA followed by the Bonferroni post hoc test. Statistical significance levels of *p < 0.05, **p < 0.01, ***p < 0.001, ****p < 0.0001 were considered significant.

## 3 RESULTS

### 3.1 Cytotoxicity of PTX and/or OLE

To determine the cytotoxic effect of OLE and PTX on the MCF-7 cell line, the cell index was determined using the xCELLigence RTCA method. At the end of 48 hours, no significant differences were observed in the cytotoxicity results of OLE and PTX compared to the control (data not shown). The effect dose at the 24^th^ hour was preferred as the treatment dose. The changes in MCF-7 cell viability with different OLE concentrations are shown in Fig. (**1A**). As shown in Fig. (**1A**), MCF-7 cells were treated with concentrations of 10, 25, 50, 100, 200, 300 and 400 µM for 24 hours. It was determined that 50 and 100 µM OLE concentrations increased the viability of the MCF-7 breast cancer cell line compared to other concentrations in the viability ratios calculated according to the cell index values. OLE concentrations of 200, 300, and 400 µM significantly inhibited cell viability compared to the control (Table **1**). The IC_50_ values of OLE MCF-7 cells were determined as 230 µM for 24 hours.

The changes in MCF-7 cell viability with different PTX concentrations are shown in Fig. (**1B**). As shown in Fig. (**1B**), MCF-7 cells were treated at concentrations of 5, 10, 15, 20, 25, 50 and 80 µM for 24 hours. Percent viability increased at a PTX concentration of 10 µM but decreased again at other concentrations (Table **2**). The IC_50_ values of PTX in MCF-7 cells were determined as 7.5 µM for 24 hours.

### 3.2 Combination Drug Effect Analysis

**Fig. (2):**
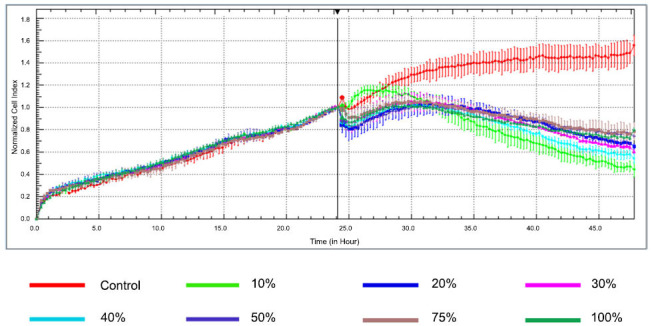
xCELLigence RTCA impedance profiles of E-plates containing MCF-7 cells treated with different concentrations (OLE+PTX) for 24 h. The different (OLE+PTX) concentrations 10% (OLE 23µM + PTX 0.75 µM) (green); 20% (OLE 46 µM+ PTX 1.5 µM) (dark blue); 30% (OLE 69 µM + PTX 2.25 µM) (pink); 40% (OLE 92 µM + PTX 3 µM) (cyan); 50% (OLE 115 µM + PTX 3.75 µM) (purple); 75% (OLE 172.5 µM + PTX 5.63 µM) (brown); 100% (OLE 230 µM + PTX 7.5 µM) (dark green) are color-coded. The red line represents the profile of the control.

Combination doses were prepared according to the IC_50_ values of OLE and PTX (Fig. **2**). The % inhibition rates of OLE and PTX combinations were determined using cell index data. According to the combinations formed at different percentages, the highest inhibition rate was 67.57% in the 10% (OLE 23 µM + PTX 0.75 µM) combination group. The combination rates applied to the MCF-7 cell line significantly reduced cell viability compared to the control group (Table **3**).

#### 3.2.1 Combination Index of OLE and PTX

**Fig. (3):**
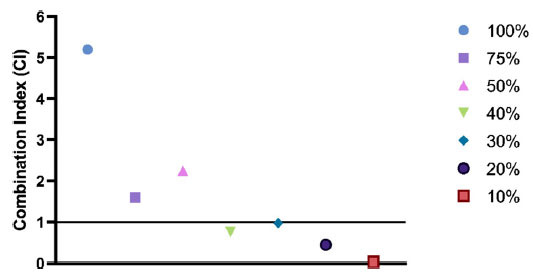
Combination indices calculated using the cytotoxicity data of OLE and PTX IC_50_ doses and their combinations at different percentages. (CI > 1 antagonism, CI = 1 additive, CI < 1 antagonism).

The CI was calculated based on the cytotoxic effects of PTX, OLE, and combinations of these two substances in different percentages on the MCF-7 breast cancer cell line obtained by the xCELLigence RTCA system. CompuSyn Software was used to determine the synergistic and antagonistic effects. The effects of 10% (OLE 23 µM+PTX 0.75 µM), 20% (OLE 46 µM+PTX 1.5 µM), 30% (OLE 69 µM+PTX 2.25 µM) and 40% (OLE 92 µM +PTX 3 µM) combination ratios, which were determined to be synergistic. The experiments were continued by selecting four combinations (Fig. **3**).

### 3.3 Oxidative Balance Status

#### 3.3.1 TOS/TAS Levels

Changes in total oxidant and antioxidant levels according to OLE and PTX IC_50_ doses and synergistic effective combination groups in MCF-7 cells are shown in Table **4**.

A statistically significant decrease was found between the TOS value of MCF-7 cells treated with OLE, PTX IC_50_ dose (6.11±1.11; 10.00±0.97 µmol/L, respectively) and the TOS value of the control group (33.33±4.20 µmol/L). The same effect was observed in the combination groups prepared with lower concentrations of OLE and PTX IC_50_ dose. Statistically significant differences were found between the TOS values of MCF-7 cells treated with 10%, 20%, 30%, and 40% (6.67±0.96; 5.56±1.47; 3.33±0.96; 13.33±2.54 µmol/L, respectively) combination rates and the TOS values of the control group. The significant decrease in TOS level in the combination group was 30% (69 µM OLE + 2.25 µM PTX).

A statistically significant increase was found between the TAS value of MCF-7 cells treated with OLE, PTX IC_50_ dose (2783.3±16.67; 1066.7±101.4 µmol/L, respectively) and the TAS value of the control group (650±50 µmol/L). This increase was more pronounced at the OLE IC_50_ dose. It was determined that there was a statistically significant increase between the TAS values of MCF-7 cells treated with 10%, 20%, 30%, and 40% (1566.7±120.2; 2200±115.5; 2716.7±60.09; 2216.6±116.7 µmol/L, respectively) combination ratios and the TAS value of the control group. The significant increase in TAS level in the combination group was 30% (69 µM OLE + 2.25 µM PTX).

#### 3.3.2 OSI

Statistically significant decreases were found between OLE, PTX IC_50_ dose, and the OSI values of the combination and the control groups. A significant decrease in the OSI level in the combination group was 30% (Table **5**).

### 3.4 SOD Activity

**Fig. (4):**
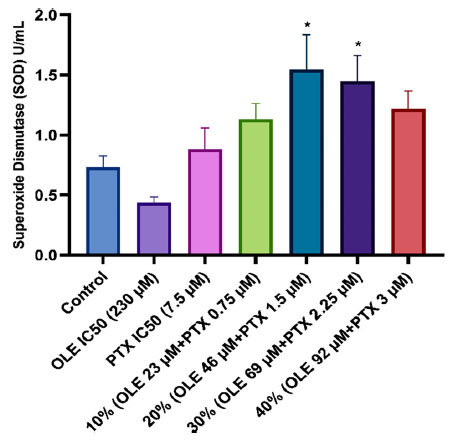
SOD activity in MCF-7 cells treated with OLE, PTX IC_50,_ and their combination groups. *p < 0.05 when compared with the control. The results are shown as mean ± SEM of three experiments (n = 3).

It was determined that the SOD activity of MCF-7 cells treated with 20% and 30% (1.542±0.29 U/mL; 1.445±0.22 U/mL, respectively) combination ratios statistically significantly increased compared to the control group (0.733±0.09 U/mL). The SOD activity of MCF-7 cells treated with the OLE IC_50_ dose (0.436±0.05 U/mL) decreased compared to the SOD activity of the control group; however, this decrease was not statistically significant. It was determined that the calculated SOD activities of PTX IC_50_ dose, 10% and 40% (0.882±0.18; 1.132±0.13 and 1.219±0.15 U/mL, respectively) combination ratios increased compared to the SOD activity of the control group; however, this increase was not statistically significant (Fig. **4**).

### 3.5 GSH Levels

**Fig. (5):**
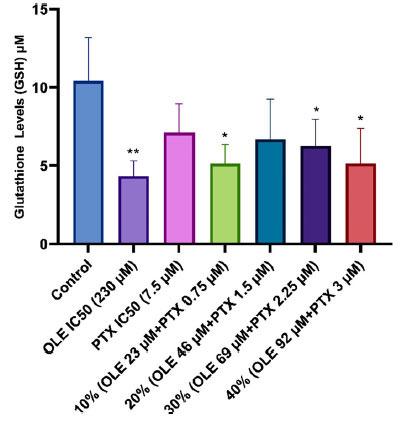
GSH levels in MCF-7 cells treated with OLE, PTX IC_50,_ and their combination groups. *p < 0.05, **p < 0.01 when compared with the control. The results are shown as mean ± SEM of three experiments (n = 3).

Compared to the control group (10.413±1.43 µM) it was determined that the GSH activity of MCF-7 cells treated with OLE IC_50_ dose, 10% and 30% combination ratios (4.318±0.98; 5.123±1.24; 6.255±1.71 µM, respectively) statistically significantly decreased. It was determined that the calculated GSH activities of PTX IC_50_ dose, 20% and 40% combination rates (7.097±1.84; 6.679±2.57; 5.128±2.26 µM, respectively) decreased compared to the GSH activity of the control group; however, this decrease was not statistically significant (Fig. **5**).

### 3.6 MDA Levels

**Fig. (6):**
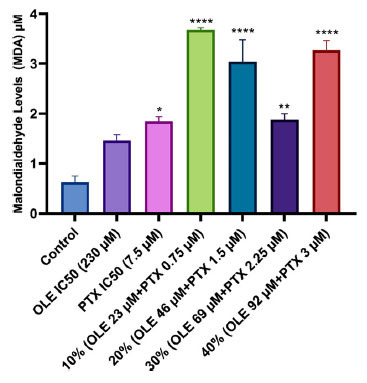
MDA activity in MCF-7 cells treated with OLE, PTX IC_50_ and their combination groups. *p < 0.05, **p < 0.01, ****p < 0.0001 when compared with the control. The results are shown as mean ± SEM of three experiments (n = 3).

A statistically significant increase was found between the MDA activity of MCF-7 cells treated with PTX IC_50_ dose, 10%, 20%, 30%, and 40% (1.667±0.71; 3.682±0.39, 2.708±0.45, 1.875±0.12 and 3.165±0.20 µM, respectively) combination ratios and the MDA activity of the control group (0.625±0.12 µM). Compared to the control group, the increase in MCF-7 cells treated with the OLE IC_50_ dose (1.458±0.13 µM) was not statistically significant (Fig. **6**).

## 4 DISCUSSION

Many chemotherapeutic agents have been used in the treatment of breast cancer, and the most important effect expected from these agents is the destruction of the signaling pathways involved in the basic processes of cancer cells, such as growth, survival, and differentiation [43]. However, the use of PTX in cancer treatment is limited owing to various side effects. PTX hypersensitivity may manifest as side effects, such as dyspnea, bronchospasm, urticaria, flushing, erythematous rash, hypertension, angioedema, chest pain, abdominal pain, fever, or chills within the first ten minutes after drug administration [44, 20]. Therefore, it is extremely important to develop the properties of cancer cells to reduce their resistance to drugs, increase their effectiveness in cancer treatment, and reduce the side effects caused by their use [45, 46]. In addition, the effect of chemotherapeutic agents on normal cells during cancer treatment may cause adverse conditions in patients. It is therefore vital to minimize damage to normal cells and limit side effects. Several *in vitro* and *in vivo* studies have suggested that the combined use of natural polyphenols with chemotherapeutics may offer increased efficacy in cancer treatment, reduced side effects, and improved effective strategies against resistant cancer cells [47, 48]. These findings suggest that natural polyphenols may offer a promising approach to enhancing cancer treatment outcomes. One of these natural compounds, OLE, is a natural phenolic compound that is abundant in olive leaves and fruits and is responsible for the bitter taste of olives. A study by Escrich *et al*. investigated how diets rich in extra virgin olive oil reduced the risk of breast cancer compared with diets rich in corn oil. Olive oil causes molecular changes in tumors, resulting in higher apoptosis rates, lower proliferation, and lower DNA damage [49]. OLE, abundant in olive leaves, is a natural phenolic compound with beneficial health effects including anticancer, antioxidant, antidiabetic, cardioprotective, and neuroprotective effects [50, 51]. Many studies have shown that OLE affects gene expression in the proliferation, apoptosis, and differentiation of cancer cells [52, 53]. In addition, studies have shown that OLE leads to an increase in the antioxidant response, can provide adequate protection against peroxidation, and prevents oxidative stress by scavenging free radical species [54, 55]. In studies on normal breast and lung cells, it was reported that OLE did not have a significant cytotoxic effect on normal cells [56, 57]. Based on the results of the literature review, this is the first study aiming to investigate the oxidative effect of OLE and PTX combination on MCF-7 breast cancer cells.

In the cytotoxicity study of OLE and PTX using the xCELLigence real-time analysis system, the IC_50_ values for PTX and OLE in MCF-7 cells treated for 24 hours were determined to be 7.5 µM and 230 µM respectively. In a report by Przychodzen *et al*., the 24-hour IC_50_ dose of OLE was determined to be 90 µM using the MTT assay [58]. In another study, the antiproliferative and apoptotic effects of OLE were investigated, and it was reported that it reduced cell viability and stimulated apoptosis in MCF-7 breast cancer cells and the IC_50_ dose of OLE was found to be 200 µg/mL in a cytotoxicity study performed using the MTT assay [59]. Within the scope of our study, cytotoxic effects were measured using the xCELLigence real-time analysis system. Using this analysis method, situations that may occur with respect to the accuracy of the experimental data were minimized. Siriani *et al*. reported that OLE may have a chemopreventive role in cell proliferation in MCF-7 (estrogen receptor-positive) cells through the inhibition of estrogen-dependent fast signals involved in cell growth [60]. In addition, *in vivo* studies have reported that OLE slows tumor growth and inhibits metastasis [61, 62].

The cytotoxic effect of OLE and PTX combinations on MCF-7 cells using the xCELLigence RTCA system showed that 10%, 20%, 30%, and 40% combinations had a synergistic effect (Fig. **3**). According to our results, co-administration of OLE and PTX to MCF-7 cells reduced PTX IC_50_ dose compared to PTX alone. Our findings demonstrate that both OLE and PTX are cytotoxic to MCF-7 cells and that simultaneous treatment with PTX-OLE elicits a synergistic effect. Different studies have reported the possible outcomes of different chemotherapeutic drugs in combination with OLE. In a study by Choupani *et al*., a combination of doxorubicin (DOX), a chemotherapeutic agent used in the treatment of breast cancer, and OLE was applied to MCF-7 cells. DOX used in combination with OLE has been shown to have greater cytotoxic and apoptotic effects than DOX alone [63]. Furthermore, an *in vivo* study showed that OLE enhanced tumor apoptosis induced by chemotherapeutics. In nude mice bearing MDA-MB-231 xenografts, OLE (50 mg/kg) combined with DOX (2.5 mg/kg) induced apoptosis *via* the mitochondrial pathway [64]. Therefore, more detailed studies on the use of OLE in combination therapies are needed. However, in addition to these studies, the side effects and safety of the plant-based approach are important issues in terms of the preferability of such combination therapies. It should be considered that each herbal preparation may have different effects on different individuals. Therefore, there is a need for more detailed *in vivo* studies of herbal preparations in the future.

ROS are normally produced by cells and are maintained in balance with antioxidant defense; however, disruption of this balance and excessive ROS accumulation causes oxidative stress. Oxidative stress is harmful to cells and can trigger many diseases, including cancer. In this study, in which we measured TAS levels to determine the level of antioxidants produced by the cell against free radicals, the TAS levels for 10%, 20%, 30%, and 40% combinations of OLE with PTX increased significantly compared to the control group (Table **4**). The effect of OLE on TAS levels may be due to its strong antioxidant properties. OLE protects cellular macromolecules from oxidative damage *via* its antioxidant effects [65]. In a study by El-Azem *et al*., the combination of hydroxytyrosol, one of the phenolic compounds found in olives, and the hydrolysis product of OLE, and PTX was applied to rats with breast cancer, and it was shown that this combination treatment increased the antioxidant capacity [66]. This experimental result is compatible with those of our study. According to one study, OLE reduces the level of TOS in the MCF-7 cell line [67]. Similar to this study, the results of our study on TOS provide information on the total oxidant level of the cell and show that OLE significantly reduced the TOS level compared to the control group (Table **4**). In addition, it was determined that the OSI values in the combination groups were significantly lower than in the control group. The increased TAS level resulting from the antioxidant effect of OLE and the defense mechanism against free radicals may have caused a decrease in OSI values (Table **5**). The effect on SOD activity may indirectly be an indicator of increased TAS levels. SOD is an enzymatic antioxidant that catalyzes the dismutation of harmful superoxide anions to hydrogen peroxide and molecular oxygen, forming the cells’ first line of defense against ROS. From our study, the TAS level increased and SOD activity showed a similar increase when OLE was combined with PTX. Since ROS is the primary antioxidant produced against them, an increase in SOD activity is an indicator of defense against increased free radicals. The OLE IC_50_ dose alone reduced SOD activity (Fig. **4**). This result of OLE administration alone may indicate that it affects the cells by a different mechanism; therefore, further studies are needed to determine the molecular mechanism underlying the effect of OLE alone. In a study by Arı *et al*., the application of OLE alone to MCF-7 cells decreased SOD levels compared with the control [67]. Furthermore, a study in a mouse model of breast cancer showed a reduction in breast cancer volume after treatment with 150 and 225 mg/kg/day OLE. It has been suggested that this reduced growth is related to the polyphenol content of OLE, which leads to an increase in the activity of antioxidant enzymes, including SOD and catalase [68].

One of the distinguishing attributes possessed by cancer cells is their elevated concentration of glutathione (GSH). This particular characteristic enables cancer cells to exhibit resistance against apoptosis induced by chemotherapy [69, 70]. The initiation of apoptosis mechanisms is induced by the depletion of GSH, which plays a crucial role in biological processes associated with cell survival [71]. In our investigation, the simultaneous administration of OLE and PTX resulted in a reduction of GSH activity in comparison to the control group (Fig. **5**). In this context, we believe that the combination of PTX and OLE may stimulate apoptotic mechanisms in cells. Studies have reported that a decrease in the amount of GSH, due to an increase in ROS, is an indicator of cell death [72, 73]. According to one study, the stimulation of oxidative stress in the HeLa cell line leads to GSH depletion, resulting in apoptosis and necrosis mechanisms [74].

Also, the MDA level, which is the best marker of oxidative stress that causes an increase in free radicals, was analyzed. Lipid peroxidation is a biological process induced by free radicals and normally occurs in all cells and tissues [75]. The combination of OLE and PTX increased the MDA level significantly compared to the control group (Fig. **6**). The increase in MDA may have been a result of oxidative stress due to an increase in ROS. *In vivo*, studies have reported that oxidative stress is associated with increased MDA levels, consistent with our findings [76, 77]. In studies comparing cancer patient groups and healthy control groups, MDA levels were found to be higher in patients with cancer, which is an indicator of increased ROS and oxidative stress in cancer cells [78, 79].

Considering our study and those in the literature, we concluded that combinations of OLE and PTX at doses lower than the IC_50_ dose may decrease cell proliferation regarding oxidative stress. However, the molecular pathways that may be related to the decrease in cell proliferation were not examined in this study. In future studies, we plan to examine the relationship between the results obtained, molecular mechanisms, and death pathways. In particular, investigating how and through which metabolic pathways affect cancer cell death may contribute to the development of potential therapeutic strategies. In this context, we believe that the results of this study are preliminary, and may shed light on future studies.

## CONCLUSION

In light of the data obtained, it was determined that OLE + PTX combinations formed at high doses were antagonistic (100%, 75%, 50%) in the MCF-7 cell line, but the combinations applied at low doses had a synergistic effect (40%, 30%, 20%, 10%). It was observed that the dose of PTX in the combination groups found to be synergistic was lower than the IC_50_ (7.5 µM) dose of PTX alone in the MCF-7 cell line. The combined application of OLE and PTX reduced the toxic doses of PTX. In terms of the combined use of OLE and PTX on oxidative stress, antioxidant defense increased and OSI levels decreased. It was determined that the SOD level in the combination groups increased according to the OLE and PTX IC_50_ dose applications. It was observed that the GSH level of the combination groups decreased according to the application of the PTX IC_50_ dose. In addition, it was determined that the combination groups increased the MDA level. Further studies are needed to answer questions such as how the effects of the combinations in the MCF-7 cell line occur, through which specific pathways, and whether the combinations are effective *in vivo*.

## Figures and Tables

**Fig. (1):**
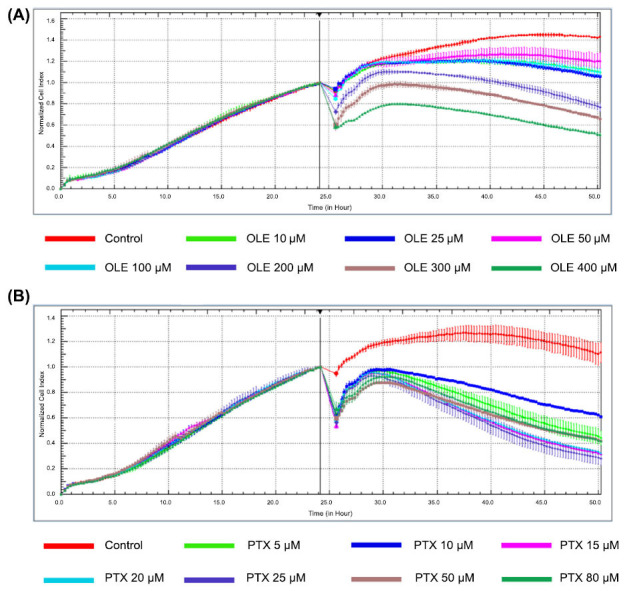
xCELLigence RTCA impedance profiles of E-plates containing MCF-7 cells treated with different concentrations of OLE (**A**) and PTX (**B**) for 24 hours.

**Table 1:** Viability rates measured by the xCELLigence RTCA method after 24 hours treatment of MCF-7 cells with different OLE concentrations.

**OLE Concentration (µM)**	**Viability (%)**
Control (0 µM)	100
10 µM	80.39 ± 3.51**
25 µM	84.71 ± 6.41*
50 µM	98.06 ± 7.44
100 µM	92.59 ± 7.19
200 µM	54.59 ± 0.04****
300 µM	47.36 ± 3.67****
400 µM	32.03 ± 1.07****

**Note:** *p < 0.05, **p < 0.01, ****p < 0.0001 when compared with the control.

**Table 2:** Viability rates measured by the xCELLigence RTCA method after 24 hours of treatment of MCF-7 cells with different PTX concentrations.

**PTX Concentration (µM)**	**Viability (%)**
Control (0 µM)	100
5 µM	46.64 ± 0.42**
10 µM	61.62 ± 0.49**
15 µM	37.25 ± 0.48**
20 µM	38.33 ± 0.13**
25 µM	31.78 ± 1.82**
50 µM	41.02 ± 0.12**
80 µM	37.65 ± 0.17**

**Note:** **p < 0.01 when compared with the control.

**Table 3:** Inhibition rates measured by the xCELLigence RTCA method after 24 hours of treatment of MCF-7 cells with different combination doses calculated over different percentages of PTX and OLE IC_50_ doses.

**% IC_50_**	**Inhibition (%)**
10% (OLE 23 µM+PTX 0.75 µM)	65.57 ± 3.19****
20% (OLE 46 µM+PTX 1.5 µM)	49.35 ± 0.96****
30% (OLE 69 µM+PTX 2.25 µM)	46.18 ± 1.85****
40% (OLE 92 µM+PTX 3 µM)	50.72 ± 1.59****
50% (OLE 115 µM+PTX 3.75 µM)	43.56 ± 0.39****
75% (OLE 172.5 µM+PTX 5.63 µM)	49.69 ± 5.28****
100% (OLE 230 µM+PTX 7.5 µM)	42.43 ± 2.17****

**Note:** ****p < 0.0001 when compared with the control.

**Table 4:** TOS and TAS of OLE and PTX IC_50_ doses, and combinations formed at different percentages in MCF-7 cells.

**Groups**	**TOS (µmol/L)**	**TAS (µmol/L)**
Control	33.33 ± 4.20	650 ± 50
OLE IC_50_	6.11 ± 1.11****	2783.3 ± 16.67****
PTX IC_50_	10.00 ± 0.97****	1066.7 ± 101.4***
10%	6.67 ± 0.96****	1566.7 ± 120.2****
20%	5.56 ± 1.47****	2200 ± 115.5****
30%	3.33 ± 0.96****	2716.7 ± 60.09****
40%	13.33 ± 2.54***	2216.6 ± 116.7****

**Note:** ***p < 0.001, ****p < 0.0001 when compared with the control. The results are shown as mean ± SEM of three experiments (n = 3).

**Table 5:** OSI values calculated using TOS and TAS values.

**Groups**	**OSI**
Control	5.24 ± 0.92
OLE IC_50_	0.22 ± 0.04****
PTX IC_50_	0.94 ± 0.09****
10%	0.42 ± 0.05****
20%	0.26 ± 0.07****
30%	0.12 ± 0.03****
40%	0.62 ± 0.15****

**Note:** ****p < 0.0001 when compared with the control. The results are shown as mean ± SEM of three experiments (n = 3).

## Data Availability

The authors confirm that the data supporting the findings of this study are available within the article.

## LIST OF ABBREVIATIONS

CI: Combination Index
DMEM: Dulbecco’s Modified Eagle’s Medium
DMSO: Dimethyl Sulfoxide
DOX: Doxorubicin
FBS: Fetal Bovine Serum
GSH: Glutathione
MDA: Malondialdehyde
OLE: Oleuropein
OSI: Oxidative Stress Index
PTX: Paclitaxel
ROS: Reactive Oxygen Species
RTCA: xCELLigence Real-time Cell Analyzer
SEM: Standard Error
SOD: Superoxide Dismutase
TAS: Total Antioxidant Status
TOS: Total Oxidant Status

## References

[1] Smolarz B., Nowak A.Z., Romanowicz H. (2022). Breast cancer—epidemiology, classification, pathogenesis and treatment (Review of Literature).. Cancers.

[2] Sung H., Ferlay J., Siegel R.L., Laversanne M., Soerjomataram I., Jemal A., Bray F. (2021). Global Cancer Statistics 2020: GLOBOCAN estimates of incidence and mortality worldwide for 36 cancers in 185 countries.. CA Cancer J. Clin..

[3] Nguyen L.H., Goel A., Chung D.C. (2020). Pathways of colorectal carcinogenesis.. Gastroenterology.

[4] Peters J.M., Gonzalez F.J. (2018). The evolution of carcinogenesis.. Toxicol. Sci..

[5] Agrawal K., Asthana S., Kumar D. (2023). Role of oxidative stress in metabolic reprogramming of brain cancer.. Cancers.

[6] Liu L., Hou Q., Chen B., Lai X., Wang H., Liu H., Wu L., Liu S., Luo K., Liu J. (2023). Identification of molecular subgroups and establishment of risk model based on the response to oxidative stress to predict overall survival of patients with lung adenocarcinoma.. Eur. J. Med. Res..

[7] Peña-Oyarzun D., Bravo-Sagua R., Diaz-Vega A., Aleman L., Chiong M., Garcia L., Bambs C., Troncoso R., Cifuentes M., Morselli E., Ferreccio C., Quest A.F.G., Criollo A., Lavandero S. (2018). Autophagy and oxidative stress in non-communicable diseases: A matter of the inflammatory state?. Free Radic. Biol. Med..

[8] Georgescu S.R., Mitran C.I., Mitran M.I., Caruntu C., Sarbu M.I., Matei C., Nicolae I., Tocut S.M., Popa M.I., Tampa M. (2018). New insights in the pathogenesis of hpv infection and the associated carcinogenic processes: The role of chronic inflammation and oxidative stress.. J. Immunol. Res..

[9] Sosa V., Moliné T., Somoza R., Paciucci R., Kondoh H. (2013). LLeonart, M.E. Oxidative stress and cancer: An overview.. Ageing Res. Rev..

[10] Azmanova M., Pitto-Barry A. (2022). Oxidative stress in cancer therapy: Friend or enemy?. ChemBioChem.

[11] Moo T.A., Sanford R., Dang C., Morrow M. (2018). Overview of breast cancer therapy.. PET Clin..

[12] Ye F., Dewanjee S., Li Y., Jha N.K., Chen Z-S., Kumar A. (2023). Advancements in clinical aspects of targeted therapy and immunotherapy in breast cancer.. Mol. Cancer.

[13] Pons D.G., Nadal-Serrano M., Torrens-Mas M., Valle A., Oliver J., Roca P. (2015). UCP2 inhibition sensitizes breast cancer cells to therapeutic agents by increasing oxidative stress.. Free Radic. Biol. Med..

[14] Lewis-Wambi J.S., Kim H.R., Wambi C., Patel R., Pyle J.R., Klein-Szanto A.J., Jordan V.C. (2008). Buthionine sulfoximine sensitizes antihormone-resistant human breast cancer cells to estrogen-induced apoptosis.. Breast Cancer Res..

[15] Wani M.C., Taylor H.L., Wall M.E., Coggon P., McPhail A.T. (1971). Plant antitumor agents. VI. Isolation and structure of taxol, a novel antileukemic and antitumor agent from Taxus brevifolia.. J. Am. Chem. Soc..

[16] Foley E.A., Kapoor T.M. (2013). Microtubule attachment and spindle assembly checkpoint signalling at the kinetochore.. Nat. Rev. Mol. Cell Biol..

[17] Liao P.C., Lieu C.H. (2005). Cell cycle specific induction of apoptosis and necrosis by paclitaxel in the leukemic U937 cells.. Life Sci..

[18] Varbiro G., Veres B., Gallyas F., Sumegi B. (2001). Direct effect of Taxol on free radical formation and mitochondrial permeability transition.. Free Radic. Biol. Med..

[19] Yamamoto Y., Kawano I., Iwase H. (2011). Nab-paclitaxel for the treatment of breast cancer: Efficacy, safety, and approval.. OncoTargets Ther..

[20] Vishnu P., Roy V. (2011). Safety and efficacy of nab -paclitaxel in the treatment of patients with breast cancer.. Breast Cancer.

[21] Yue Q.X., Liu X., Guo D.A. (2010). Microtubule-binding natural products for cancer therapy.. Planta Med..

[22] Li W.B., Li Y., Yu C., He Y.M. (2015). Reversal of multidrug resistance by the chinese medicine yiqi jianpi huaji decoction and the mechanism of action in human gastric cancer SGC7901/VCR cells.. Evid. Based Complement. Alternat. Med..

[23] Bach-Faig A., Berry E.M., Lairon D., Reguant J., Trichopoulou A., Dernini S., Medina F.X., Battino M., Belahsen R., Miranda G., Serra-Majem L. (2011). Mediterranean diet pyramid today. Science and cultural updates.. Public Health Nutr..

[24] García-Segovia P., Sánchez-Villegas A., Doreste J., Santana F., Serra-Majem L. (2006). Olive oil consumption and risk of breast cancer in the Canary Islands: A population-based case–control study.. Public Health Nutr..

[25] La Vecchia C., Negri E., Franceschi S., Decarli A., Giacosa A., Lipworth L. (1995). Olive oil, other dietary fats, and the risk of breast cancer (Italy).. Cancer Causes Control.

[26] de Lorgeril M., Salen P., Martin J.L., Monjaud I., Boucher P., Mamelle N. (1998). Mediterranean dietary pattern in a randomized trial: Prolonged survival and possible reduced cancer rate.. Arch. Intern. Med..

[27] Nediani C., Ruzzolini J., Romani A., Calorini L. (2019). Oleuropein, a bioactive compound from Olea europaea L., as a potential preventive and therapeutic agent in non-communicable diseases.. Antioxidants.

[28] Martínez-González M.A., Sayón-Orea C., Bullón-Vela V., Bes-Rastrollo M., Rodríguez-Artalejo F., Yusta-Boyo M.J., García-Solano M. (2022). Effect of olive oil consumption on cardiovascular disease, cancer, type 2 diabetes, and all-cause mortality: A systematic review and meta-analysis.. Clin. Nutr..

[29] Moral R., Escrich E. (2022). Influence of olive oil and its components on breast cancer: Molecular mechanisms.. Molecules.

[30] Mitra S., Dash R. (2018). Natural products for the management and prevention of breast cancer.. Evid. Based Complement. Alternat. Med..

[31] Sznarkowska A., Kostecka A., Meller K., Bielawski K.P. (2017). Inhibition of cancer antioxidant defense by natural compounds.. Oncotarget.

[32] Panieri E., Santoro M.M. (2016). ROS homeostasis and metabolism: A dangerous liason in cancer cells.. Cell Death Dis..

[33] Gikas E., Bazoti F.N., Tsarbopoulos A. (2007). Conformation of oleuropein, the major bioactive compound of Olea europea.. J. Mol. Struct. Theochem..

[34] Servili M., Esposto S., Fabiani R., Urbani S., Taticchi A., Mariucci F., Selvaggini R., Montedoro G.F. (2009). Phenolic compounds in olive oil: Antioxidant, health and organoleptic activities according to their chemical structure.. Inflammopharmacology.

[35] Cicerale S., Lucas L.J., Keast R.S.J. (2012). Antimicrobial, antioxidant and anti-inflammatory phenolic activities in extra virgin olive oil.. Curr. Opin. Biotechnol..

[36] Piroddi M., Albini A., Fabiani R., Giovannelli L., Luceri C., Natella F., Rosignoli P., Rossi T., Taticchi A., Servili M., Galli F. (2017). Nutrigenomics of extra‐virgin olive oil: A review.. Biofactors.

[37] Delboccio P., Dideo A., Decurtis A., Celli N., Iacoviello L., Rotilio D. (2003). Liquid chromatography–tandem mass spectrometry analysis of oleuropein and its metabolite hydroxytyrosol in rat plasma and urine after oral administration.. J. Chromatogr. B Analyt. Technol. Biomed. Life Sci..

[38] Kimura Y., Sumiyoshi M. (2009). Olive leaf extract and its main component oleuropein prevent chronic ultraviolet B radiation-induced skin damage and carcinogenesis in hairless mice.. J. Nutr..

[39] Chou T.C., Talalay P. (1984). Quantitative analysis of dose-effect relationships: the combined effects of multiple drugs or enzyme inhibitors.. Adv. Enzyme Regul..

[40] Chou T.C. (2006). Theoretical basis, experimental design, and computerized simulation of synergism and antagonism in drug combination studies.. Pharmacol. Rev..

[41] Erel O. (2005). A new automated colorimetric method for measuring total oxidant status.. Clin. Biochem..

[42] Erel O. (2004). A novel automated direct measurement method for total antioxidant capacity using a new generation, more stable ABTS radical cation.. Clin. Biochem..

[43] Altmann K.H., Gertsch J. (2007). Anticancer drugs from nature—natural products as a unique source of new microtubule-stabilizing agents.. Nat. Prod. Rep..

[44] Haddad R., Alrabadi N., Altaani B., Li T. (2022). Paclitaxel drug delivery systems: Focus on Nanocrystals’ surface modifications.. Polymers.

[45] Sparano J.A., Wang M., Martino S., Jones V., Perez E.A., Saphner T., Wolff A.C., Sledge G.W., Wood W.C., Davidson N.E. (2008). Weekly paclitaxel in the adjuvant treatment of breast cancer.. N. Engl. J. Med..

[46] Amjad M.T., Chidharla A., Kasi A. (2022). Cancer Chemotherapy.. StatPearls..

[47] Abu Samaan T.M., Samec M., Liskova A., Kubatka P., Büsselberg D. (2019). Paclitaxel’s mechanistic and clinical effects on breast cancer.. Biomolecules.

[48] Fantini M., Benvenuto M., Masuelli L., Frajese G., Tresoldi I., Modesti A., Bei R. (2015). In vitro and in vivo antitumoral effects of combinations of polyphenols, or polyphenols and anticancer drugs: perspectives on cancer treatment.. Int. J. Mol. Sci..

[49] Nurgali K., Jagoe R.T., Abalo R. (2018). Editorial: Adverse effects of cancer chemotherapy: Anything new to improve tolerance and reduce sequelae?. Front. Pharmacol..

[50] Escrich E., Moral R., Solanas M. (2011). Olive oil, an essential component of the Mediterranean diet, and breast cancer.. Public Health Nutr..

[51] Gorzynik-Debicka M., Przychodzen P., Cappello F., Kuban-Jankowska A., Marino G.A., Knap N., Wozniak M., Gorska-Ponikowska M. (2018). Potential health benefits of olive oil and plant polyphenols.. Int. J. Mol. Sci..

[52] Nenadis N., Papoti V.T., Tsimidou M.Z. (2021). Bioactive ingredients in olive leaves.. Olives and Olive Oil in Health and Disease Prevention.

[53] Di Francesco A., Falconi A., Di Germanio C., Micioni Di Bonaventura M.V., Costa A., Caramuta S., Del Carlo M., Compagnone D., Dainese E., Cifani C., Maccarrone M., D’Addario C. (2015). Extravirgin olive oil up-regulates CB1 tumor suppressor gene in human colon cancer cells and in rat colon via epigenetic mechanisms.. J. Nutr. Biochem..

[54] Hassan Z.K., Elamin M.H., Daghestani M.H., Omer S.A., Al-Olayan E.M., Elobeid M.A., Virk P., Mohammed O.B. (2012). Oleuropein induces anti-metastatic effects in breast cancer.. Asian Pac. J. Cancer Prev..

[55] (2015). Žukovec Topalović D.; Živković L.; Čabarkapa, A.; Djelić N.; Bajić V.; Dekanski, D.; Spremo-Potparević B. Dry olive leaf extract counteracts L-thyroxine-induced genotoxicity in human peripheral blood leukocytes in vitro.. Oxid. Med. Cell. Longev..

[56] Visioli F., Galli C. (2002). Biological properties of olive oil phytochemicals.. Crit. Rev. Food Sci. Nutr..

[57] Cao S., Zhu X., Du L. (2017). P38 MAP kinase is involved in oleuropein-induced apoptosis in A549 cells by a mitochondrial apoptotic cascade.. Biomed. Pharmacother..

[58] Elamin M.H., Daghestani M.H., Omer S.A., Elobeid M.A., Virk P., Al-Olayan E.M., Hassan Z.K., Mohammed O.B., Aboussekhra A. (2013). Olive oil oleuropein has anti-breast cancer properties with higher efficiency on ER-negative cells.. Food Chem. Toxicol..

[59] Przychodzen P., Kuban-Jankowska A., Wyszkowska R., Barone G., Bosco G.L., Celso F.L., Kamm A., Daca A., Kostrzewa T., Gorska-Ponikowska M. (2019). PTP1B phosphatase as a novel target of oleuropein activity in MCF-7 breast cancer model.. Toxicol. In Vitro.

[60] Sirianni R., Chimento A., De Luca A., Casaburi I., Rizza P., Onofrio A., Iacopetta D., Puoci F., Andò S., Maggiolini M., Pezzi V. (2010). Oleuropein and hydroxytyrosol inhibit MCF‐7 breast cancer cell proliferation interfering with ERK1/2 activation.. Mol. Nutr. Food Res..

[61] Han J., Talorete T.P.N., Yamada P., Isoda H. (2009). Anti-proliferative and apoptotic effects of oleuropein and hydroxytyrosol on human breast cancer MCF-7 cells.. Cytotechnology.

[62] Sepporta M.V., Fuccelli R., Rosignoli P., Ricci G., Servili M., Morozzi G., Fabiani R. (2014). Oleuropein inhibits tumour growth and metastases dissemination in ovariectomised nude mice with MCF-7 human breast tumour xenografts.. J. Funct. Foods.

[63] Milanizadeh S., Bigdeli M.R., Rasoulian B., Amani D. (2014). The effects of olive leaf extract on antioxidant enzymes activity and tumor growth in breast cancer.. Thrita.

[64] Choupani J., Alivand M.R., Derakhshan M.S., Zaeifizadeh M., S. Khaniani M. (2019). Oleuropein inhibits migration ability through suppression of epithelial-mesenchymal transition and synergistically enhances doxorubicin-mediated apoptosis in MCF-7 cells.. J. Cell. Physiol..

[65] Barbaro B., Toietta G., Maggio R., Arciello M., Tarocchi M., Galli A., Balsano C. (2014). Effects of the olive-derived polyphenol oleuropein on human health.. Int. J. Mol. Sci..

[66] El-azem N., Pulido-Moran M., Ramirez-Tortosa C.L., Quiles J.L., Cara F.E., Sanchez-Rovira P., Granados-Principal S., Ramirez-Tortosa M.C. (2019). Modulation by hydroxytyrosol of oxidative stress and antitumor activities of paclitaxel in breast cancer.. Eur. J. Nutr..

[67] (2018). Arı M.; Karul, A.; Sakarya, S. Investigation of antiproliferative, apoptotic and antioxidant effects of oleuropein and vitamin D on breast cancer cell lines (MCF-7).. Proceedings.

[68] Milanizadeh S., Reza B.M. (2019). Pro-apoptotic and anti-angiogenesis effects of olive leaf extract on spontaneous mouse mammary tumor model by balancing vascular endothelial growth factor and endostatin levels.. Nutr. Cancer.

[69] Dalton T.P., Chen Y., Schneider S.N., Nebert D.W., Shertzer H.G. (2004). Genetically altered mice to evaluate glutathione homeostasis in health and disease.. Free Radic. Biol. Med..

[70] Estrela J.M., Ortega A., Obrador E. (2006). Glutathione in cancer biology and therapy.. Crit. Rev. Clin. Lab. Sci..

[71] Franco R., Cidlowski J.A. (2009). Apoptosis and glutathione: Beyond an antioxidant.. Cell Death Differ..

[72] Ortega A.L., Mena S., Estrela J.M. (2011). Glutathione in cancer cell death.. Cancers.

[73] Cui X.Y., Park S.H., Park W.H. (2022). Anti-cancer effects of auranofin in human lung cancer cells by increasing intracellular ROS levels and depleting GSH levels.. Molecules.

[74] You B.R., Kim S.Z., Kim S.H., Park W.H. (2011). Gallic acid-induced lung cancer cell death is accompanied by ROS increase and glutathione depletion.. Mol. Cell. Biochem..

[75] Niki E. (2012). Do antioxidants impair signaling by reactive oxygen species and lipid oxidation products?. FEBS Lett..

[76] Celep A.G.S., Yilmaz S., Coruh N. (2012). Antioxidant capacity and cytotoxicity of Aesculus hippocastanum on breast cancer MCF-7 cells.. Yao Wu Shi Pin Fen Xi.

[77] Timur M., Akbas S.H., Ozben T. (2005). The effect of Topotecan on oxidative stress in MCF-7 human breast cancer cell line.. Acta Biochim. Pol..

[78] Wang C., Yu J., Wang H., Zhang J., Wu N. (2014). Lipid peroxidation and altered anti-oxidant status in breast adenocarcinoma patients.. Drug Res..

[79] de Oliveira S.T., Bessani M.P., Scandolara T.B., Silva J.C., Kawassaki A.C.B., Fagotti P.A.F., Maito V.T., de Souza J.A., Rech D., Panis C. (2022). Systemic lipid peroxidation profile from patients with breast cancer changes according to the lymph nodal metastasis status.. Oncoscience.

